# Exploring the association between weight-adjusted-waist index and overactive bladder: A population-based study

**DOI:** 10.1097/MD.0000000000048763

**Published:** 2026-05-08

**Authors:** Xiaoyan Hu, Weixing Jing, Yiqi Huang, Tianxiao Fu, Weigang Shen, Weicheng Xiao

**Affiliations:** a Department of Intensive Care Unit, Shaoxing Second Hospital, Shaoxing, Zhejiang, China; b Department of Emergency, Shaoxing Second Hospital, Shaoxing, Zhejiang, China; c Department of Nephrology, Shaoxing Second Hospital, Shaoxing, Zhejiang, China; d Department of Traditional Chinese Medicine, The First Affiliated Hospital of Zhejiang University, Hangzhou, Zhejiang, China; e Department of Blood Purifying Center, Shaoxing Second Hospital, Shaoxing, Zhejiang, China.

**Keywords:** abdominal obesity, NHANES, obesity, overactive bladder, weight-adjusted-waist index

## Abstract

Numerous studies have shown that obesity is a risk factor for overactive bladder (OAB). However, traditional anthropometric measures, such as body mass index and waist circumference, are limited in assessing obesity, particularly central obesity. The weight-adjusted-waist index (WWI) may provide a more accurate measure of central obesity. This study aimed to explore the association between WWI and the risk of OAB. Multivariate logistic regression and restricted cubic splines were used to analyze the relationship between WWI and the risk of OAB. Subgroup interaction analyses were conducted to assess the stability of the results. Receiver operating characteristic curves were employed to evaluate the predictive ability of various anthropometric indicators for the risk of OAB. Logistic regression analysis revealed a significant positive association between WWI and the risk of OAB. After adjusting for all potential confounders, each 1-unit increase in WWI was associated with a 26% increased risk of OAB. Dose–response curve analysis showed a linear positive relationship between WWI and OAB risk. In receiver operating characteristic curve analysis, WWI demonstrated better predictive power for OAB compared to body mass index and waist circumference (area under the curve = 0.669, 95% CI = 0.662–0.677, optimal cutoff value = 11.150). This study found that an increased WWI was strongly associated with an elevated risk of OAB. WWI also proved to be a superior anthropometric indicator for assessing the risk of OAB.

## 1. Introduction

The International Continence Society and International Urogynecological Association defined overactive bladder (OAB) in 2002 as “urgency of urination, with or without urge incontinence, usually accompanied by frequent urination or nocturia.”^[1,2]^ As a component of lower urinary tract symptoms (LUTS), OAB manifests with symptoms associated with the storage phase of urine. The Epidemiology of Prolific Incontinence and Continence study, the first investigation to assess these symptoms based on the 2002 International Continence Society definitions, represents the largest population-based survey conducted in 5 countries to determine the prevalence rates of OAB, urinary incontinence, and other LUTS. The study revealed that the prevalence of OAB is approximately 11.8%, and this rate significantly increases with advancing age. In China, an estimated prevalence rate of around 6% for OAB has been reported.^[3,4]^ Although OAB is not life-threatening, it is a functional disorder that poses significant challenges for patients. Individuals with OAB not only experience symptoms that affect their daily activities and productivity but also often suffer from feelings of embarrassment and anxiety. Furthermore, the average annual direct costs associated with managing OAB range from €269 to €706 per patient per year.^[5]^ The economic burden of OAB is substantial and is expected to increase due to demographic shifts toward an aging population. In recent years, a growing body of evidence has established a strong association between OAB and various comorbidities, including metabolic syndrome (MetS),^[6]^ sex hormone deficiency,^[7]^ urinary microbiota,^[8]^ affective disorders,^[9]^ and gastrointestinal functional disorders.^[10]^ Moreover, it is important to note that OAB may exhibit distinct pathophysiological mechanisms within each of these contexts. Identifying the underlying mechanisms contributing to a patient’s experience of OAB symptoms can facilitate personalized treatment approaches and improve clinical outcomes.

The potential etiology and contributing factors of OAB are widely recognized as complex and diverse. Notably, obesity has been identified as a prevalent risk factor for LUTS, with individuals possessing a body mass index (BMI) exceeding 25 at an increased likelihood of developing OAB.^[11–13]^ However, BMI’s reliability as a metabolic health indicator has been controversial due to its inability to differentiate between body fat and lean mass, as well as between central and peripheral fat distribution.^[14]^ To address this concern, a new measure of adiposity called the “weight-adjusted-waist index” (WWI) was introduced.^[15]^ WWI considers a standardized waist circumference (WC) relative to weight and aims to establish its correlation with obesity-related diseases and mortality.^[16]^ The calculation is performed using the formula WC/weight1/2. WWI considers a standardized WC relative to weight and aims to establish its correlation with obesity-related diseases and mortality.

Currently, there is a lack of evidence to establish a definitive correlation between WWI and OAB, which makes it challenging to assess the quantitative relationship between obesity and OAB accurately. Therefore, our study aims to ascertain the association between WWI and OAB by utilizing data from the National Health and Nutrition Examination Survey (NHANES). This approach will help evaluate the potential association and assess the predictive role of WWI in determining the prevalence of OAB.

## 2. Methods

### 2.1. Study population

The NHANES is an ongoing survey conducted by the Centers for Disease Control and Prevention, and NHANES research programs are reviewed and approved by the National Health Statistics Research Council Center.^[17]^ The purpose of the project is to assess the health and nutritional status of adults and children in the United States. It involves a variety of health and nutrition surveys, and written informed consent is obtained from each participant.

Our study included participants from NHANES (2007–2020). In our analysis, we included participants who had complete information on the OAB-related questionnaires, enlisting a total of 37,970 participants. However, this number was reduced to 36,536 after excluding those with missing anthropometric data (n = 1434). In addition, missing covariate data (n = 6396) were excluded. In the final study, 30,140 participants who met the criteria were included. Figure S1, Supplemental Digital Content, illustrates the participant selection process.

### 2.2. Assessment of WWI and OAB

The outcomes of the current analysis were OAB. A meticulous exploration of the NHANES Kidney Conditions-Urology dataset was undertaken to procure insights into manifestations of OAB, encompassing urge incontinence and nocturia. The following 2 questions were used to assess the severity of urge incontinence: “During the past 12 months, have you leaked or lost control of even a small amount of urine with an urge or pressure to urinate and you couldn’t get to the toilet fast enough?” and “How frequently does this occur?” Severity of nocturia was assessed based on another question: “During the past 30 days, how many times per night did you most typically get up to urinate, from the time you went to bed at night until the time you got up in the morning?” We leveraged the table of the Criteria for Conversion of Symptom Frequencies Recorded in NHANES and Overactive Bladder Symptom Score (OABSS) scores, as utilized by Zhu et al.^[18]^ Finally, the OABSS for each subject in the NHANES was obtained by adding the nocturia score and the urge urinary incontinence (UUI) score. An individual with a total score of 3 or greater is considered to have OAB. The OABSS score calculated based on the NHANES questionnaire is provided in Table S1, Supplemental Digital Content. WWI is a WC and weight-based anthropometric measure used for assessing obesity. An increased level of obesity is indicated by a higher WWI score. Health technicians, who received training in collecting body measurements, obtained weight and WC data at the Mobile Examination Center. Each participant’s WWI was calculated as WC (cm) divided by the square root of weight (kg). In our study, WWI was considered an exposure variable.

### 2.3. Other clinical covariates

In addition to the primary outcome variables we examined, the following variables were included as covariates: age, race (White, Black, Mexican, and other), marital status (married/living with a partner, and single/divorced/widowed), educational level (less than high school, high school or equivalent, college or above), poverty-income ratio, smoking status (never, former, current), drinking status (no, former, current), recreational activity (vigorous, moderate, no activity), hypertension (no, yes), diabetes (no, yes), cardiovascular disease (CVD; no, yes), cancer (no, yes), and urinary creatinine.

### 2.4. Statistical analysis

To minimize the impact of the NHANES complex multistage sampling design, appropriate sample weights were applied for weighted analysis according to NHANES guidelines, thereby improving the accuracy of the data. Demographic characteristics are presented as the weighted mean ± standard error for continuous variables and the weighted percentage (%) for categorical variables. Baseline characteristics of the study population were evaluated using the *t* test and chi-square test. Weighted univariate and multivariate logistic regression analyses were employed to estimate the relationship between WWI and OAB. Four weighted logistic regression models were constructed: Model 1 had no variable adjustments; Model 2 was adjusted for age, sex, and race; Model 3 was adjusted for age, sex, race, poverty-income ratio, marital status, education, smoking status, drinking status, and recreational activities; and Model 4 was further adjusted for urinary creatinine, hypertension, diabetes, CVD, and cancer.

In addition, a logistic regression analysis stratified by gender was performed to explore the effect of gender on the association between WWI and OAB risk. The number of vaginal deliveries was also adjusted for in a sensitivity analysis of the female population. Furthermore, in Model 4, a weighted restricted cubic spline plot was used to reveal dose–response relationships between WWI and OAB. Stratification by age, sex, race, smoking status, drinking status, recreational activities, hypertension, diabetes, CVD, and cancer was performed, followed by interaction analyses to examine whether there were differential associations between subgroups. Receiver operating characteristic (ROC) curves were used to assess the discriminative ability of various anthropometric indicators in identifying individuals with OAB. The Delong test was applied to evaluate statistical differences between the area under the curves (AUCs) for different anthropometric indicators. All statistical analyses were conducted and processed using R software (R4.2.3; The R Foundation for Statistical Computing). A bilateral *P* value of <.05 was considered statistically significant.

## 3. Results

### 3.1. Baseline characteristics

The study involved a total of 30,140 adults from the United States, with males accounting for 49.27% and females accounting for 50.73%. The prevalence of OAB was reported in 15.36% of the participants. Table 1 presents the characteristics of the participants categorized by WWI. The WWI ranges for the first, second, third, and fourth quartiles were as follows: 8.11 to 10.48, 10.48 to 11.05, 11.05 to 11.62, and 11.62 to 15.39, respectively. Based on the WWI categories (Q1–Q4), OAB occurrence was 6.89%, 12.18%, 17.53%, and 28.17%, respectively. Detailed demographic data for all respondents can be found in Table 1.

**Table 1:** Demographic characteristics stratified by quartile of WWI (N = 30,140).

Characteristic	Total	Quartile 1 (8.11–10.48)	Quartile 2 (10.48–11.05)	Quartile 3 (11.05–11.62)	Quartile 4 (11.62–15.39)	*P* value
Age	47.02 (0.26)	36.79 (0.31)	45.62 (0.26)	51.68 (0.33)	57.14 (0.38)	<.0001
Sex						
Male	15,007 (49.27)	4570 (58.57)	4131 (53.51)	3695 (48.69)	2611 (32.23)	
Female	15,133 (50.73)	2968 (41.43)	3395 (46.49)	3839 (51.31)	4931 (67.77)	
Race						<.0001
White	12,683 (67.84)	3089 (67.16)	3071 (67.36)	3076 (66.92)	3447 (70.39)	
Black	6498 (10.49)	2183 (13.99)	1578 (9.34)	1489 (9.45)	1248 (8.49)	
Mexican	4255 (8.11)	581 (5.21)	1022 (8.19)	1297 (10.19)	1355 (9.53)	
Other	6704 (13.55)	1685 (13.64)	1855 (15.11)	1672 (13.45)	1492 (11.58)	
Marital status						<.0001
Married/living with partner	17,976 (63.62)	4064 (57.17)	4849 (69.52)	4830 (68.15)	4233 (59.65)	
Single/divorced/widowed	12,164 (36.38)	3474 (42.83)	2677 (30.48)	2704 (31.85)	3309 (40.35)	
Education level						<.0001
Less than high school	6431 (12.93)	1020 (8.71)	1401 (11.52)	1790 (14.77)	2220 (18.22)	
High school or equivalent	6884 (23.55)	1560 (19.83)	1639 (21.49)	1821 (26.08)	1864 (28.27)	
College or above	16,825 (63.52)	4958 (71.45)	4486 (66.99)	3923 (59.15)	3458 (53.51)	
PIR						<.0001
≤1.30	9179 (19.97)	2106 (19.05)	2046 (17.50)	2314 (20.14)	2713 (24.09)	
1.31–3.50	11,361 (34.83)	2635 (32.20)	2802 (32.46)	2847 (34.80)	3077 (41.33)	
>3.50	9600 (45.20)	2797 (48.74)	2678 (50.04)	2373 (45.05)	1752 (34.58)	
Recreational activity						<.0001
No activity	15,181 (43.70)	2590 (28.64)	3527 (41.35)	4126 (48.76)	4938 (60.96)	
Moderate	7857 (28.00)	1619 (22.33)	2033 (28.94)	2247 (33.42)	1958 (28.29)	
Vigorous	7102 (28.30)	3329 (49.03)	1966 (29.72)	1161 (17.81)	646 (10.76)	
Smoking status						<.0001
Never	16,794 (56.41)	4550 (62.70)	4204 (56.54)	4026 (52.16)	4014 (52.66)	
Former	7196 (24.83)	1133 (16.88)	1701 (23.84)	2128 (30.32)	2234 (30.47)	
Now	6150 (18.76)	1855 (20.41)	1621 (19.62)	1380 (17.52)	1294 (16.87)	
Drinking status						<.0001
No	3948 (9.58)	727 (7.41)	801 (8.20)	1024 (9.88)	1396 (13.86)	
Former	3847 (9.27)	532 (5.06)	809 (7.81)	1088 (11.20)	1418 (14.53)	
Now	22,345 (81.15)	6279 (87.52)	5916 (83.99)	5422 (78.92)	4728 (71.61)	
Hypertension						<.0001
No	17,742 (63.86)	6156 (84.11)	4826 (68.39)	3892 (54.78)	2868 (41.45)	
Yes	12,398 (36.14)	1382 (15.89)	2700 (31.61)	3642 (45.22)	4674 (58.55)	
Diabetes						<.0001
No	24,561 (86.08)	7241 (97.36)	6623 (91.16)	5863 (82.42)	4834 (68.80)	
Yes	5579 (13.92)	297 (2.64)	903 (8.84)	1671 (17.58)	2708 (31.20)	
CVD						<.0001
No	27,055 (92.04)	7305 (98.01)	6999 (94.42)	6627 (89.98)	6124 (83.42)	
Yes	3085 (7.96)	233 (1.99)	527 (5.58)	907 (10.02)	1418 (16.58)	
Cancer						<.0001
No	27,262 (89.86)	7207 (95.00)	6948 (90.93)	6699 (88.30)	6408 (83.44)	
Yes	2878 (10.14)	331 (5.00)	578 (9.07)	835 (11.70)	1134 (16.56)	
OAB						<.0001
No	24,053 (84.64)	6854 (93.11)	6373 (87.82)	5804 (82.47)	5022 (71.83)	
Yes	6087 (15.36)	684 (6.89)	1153 (12.18)	1730 (17.53)	2520 (28.17)	
Creatinine urine (mg/dL)	121.59 (0.88)	128.51 (1.85)	123.05 (1.47)	119.32 (1.42)	113.10 (1.30)	<.0001
BMI	29.24 (0.08)	24.87 (0.09)	28.34 (0.08)	30.86 (0.10)	34.33 (0.16)	<.0001
Waist circumference	99.72 (0.21)	85.75 (0.21)	97.12 (0.18)	105.16 (0.21)	115.44 (0.31)	<.0001

BMI = body mass index, CVD = cardiovascular disease, OAB = overactive bladder, PIR = poverty-income ratio, WWI = weight-adjusted-waist index.

### 3.2. Associations between WWI and OAB

As shown in Table 2, the weighted multivariate logistic regression analysis demonstrated a significant correlation between higher values of WWI and an increased risk of OAB. This association remained statistically significant across all models: Model 1 (OR = 2.15, 95% CI = 2.03–2.29; *P* < .0001), Model 2 (OR = 1.52, 95% CI = 1.42–1.63; *P* < .0001), and Model 3 (OR = 1.35, 95% CI = 1.26–1.45; *P* < .0001). Furthermore, even after adjusting for all covariates in Model 4, the association remained statistically significant (OR = 1.26, 95% CI = 1.18–1.35; *P* < .0001).

**Table 2:** Logistic regression analysis of WWI and OAB.

	Model 1	*P* value	Model 2	*P* value	Model 3	*P* value	Model 4	*P* value
	OR (95%CI)		OR (95%CI)		OR (95%CI)		OR (95%CI)	
WWI	2.15 (2.03–2.29)	<.0001	1.52 (1.42–1.63)	<.0001	1.35 (1.26–1.45)	<.0001	1.26 (1.18–1.35)	<.0001
Stratified by WWI quartiles								
Quartile 1	1		1		1		1	
Quartile 2	1.88 (1.59–2.21)	<.0001	1.36 (1.15–1.61)	<.001	1.28 (1.09–1.51)	.003	1.24 (1.05–1.45)	.01
Quartile 3	2.87 (2.42–3.41)	<.0001	1.61 (1.35–1.93)	<.0001	1.41 (1.19–1.68)	<.001	1.31 (1.10–1.55)	.002
Quartile 4	5.30 (4.56–6.16)	<.0001	2.37 (1.98–2.83)	<.0001	1.87 (1.57–2.22)	<.0001	1.61 (1.36–1.90)	<.0001
*P* for trend		<.0001		<.0001		<.0001		<.0001

Model 1: unadjusted. Model 2: adjusted for age, sex, and race. Model 3: adjusted for age, sex, race, marital status, education level, PIR, recreational activity, smoking status, and drinking status. Model 4: further adjusted for creatinine urine, hypertension, diabetes, CVD as well as cancer.

CI = confidence interval, CVD = cardiovascular disease, OAB = overactive bladder, OR = odds ratio, PIR = poverty-income ratio, WWI = weight-adjusted-waist index.

To further analyze the data, WWI was transformed from a continuous variable into quartiles, while controlling for confounding factors. After accounting for all potential confounders, our findings showed a significant increase in the risk of OAB in the WWI Q2 to Q4 groups compared to the reference group (WWI Q1). Specifically, the risk of OAB increased by approximately 1.24-fold, 1.32-fold, and 1.61-fold in individuals in the WWI Q2 to Q4 groups compared to those in the WWI Q1 group (Q2 vs Q1, OR = 1.24, 95% CI = 1.05–1.45, *P* = .001; Q3 vs Q1, OR = 1.31, 95% CI = 1.10–1.55, *P* = .002; Q4 vs Q1, OR = 1.61, 95% CI = 1.36–1.90, *P* < .0001).

When logistic regression analysis was performed on the risks of WWI and OAB by gender, the study showed that the risk of OAB gradually increased with the increase in WWI in both males and females (male: OR = 1.36, 95% CI = 1.21–1.51, *P* < .0001; female: OR = 1.27, 95% CI = 1.17–1.38, *P* < .0001; Table S2, Supplemental Digital Content). To mitigate the potential impact of fertility on urogenital tract structural effects, a sensitivity analysis was conducted for the female population, adjusting for the number of vaginal births. The results still showed that the risk of OAB in females increased with higher WWI (OR = 1.78, 95% CI = 1.42–2.24, *P* < .0001; Table S3, Supplemental Digital Content).

**Figure 1.**
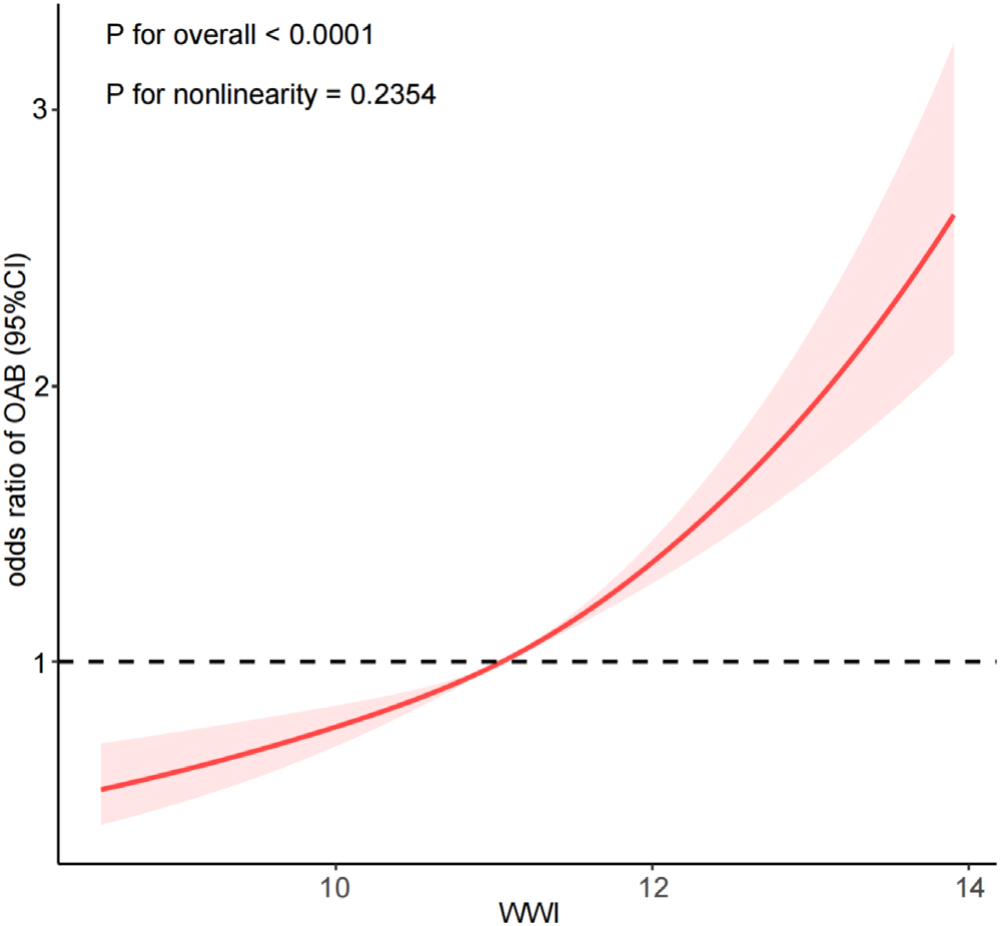
Dose–response relationship analysis between WWI and OAB. RCS regression was adjusted for age, sex, race, marital status, education level, PIR, recreational activity, smoking status, drinking status, creatinine urine, hypertension, diabetes, CVD, and cancer (Model 4). The red solid line represents ORs, red shaded region represents 95% CI. CI = confidence interval, CVD = cardiovascular disease, OAB = overactive bladder, OR = odds ratio, PIR = poverty-income ratio, RCS = restricted cubic spline, WWI = weight-adjusted-waist index.

As depicted in Figure 1, a restricted cubic spline effectively characterizes the dose–response analysis between WWI and OAB. Our findings reveal a linear positive correlation with the prevalence of OAB as WWI increases (*P* for overall <.0001; *P* for nonlinearity = .2354). Specifically, the risk of OAB escalates rapidly with higher WWI values. Upon stratifying by gender (Fig. S2, Supplemental Digital Content), we observe a significant positive association between WWI and OAB in both males and females (male: *P* for overall <.0001; *P* for nonlinearity = .0119; female: *P* for overall <.0001; *P* for nonlinearity = .4254).

**Figure 2.**
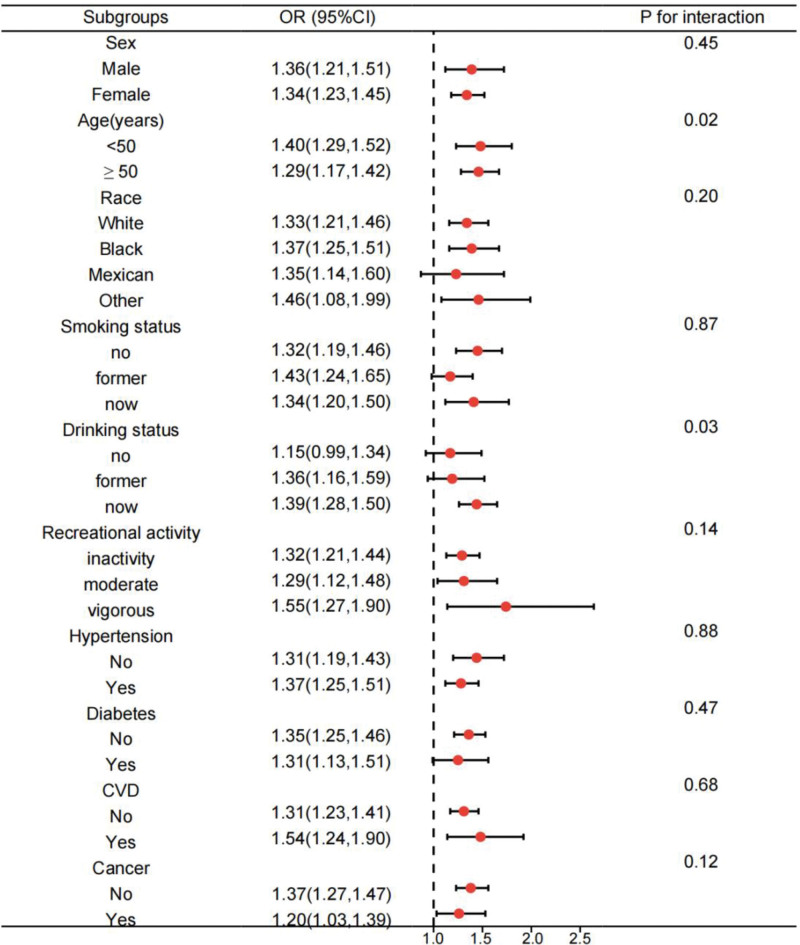
Subgroup analysis for the relationship between WWI and OAB. Analyses were adjusted for age, sex, race, marital status, education level, PIR, recreational activity, smoking status, drinking status, creatinine urine, hypertension, diabetes, CVD, and cancer. CI = confidence interval, CVD = cardiovascular disease, OAB = overactive bladder, OR = odds ratio, PIR = poverty-income ratio, WWI = weight-adjusted-waist index.

To assess the consistency of the relationship between WWI and OAB across different groups, subgroup analyses were conducted (Fig. 2) considering various factors such as gender (male or female), age (<50 or ≥50), race (White, Black, Mexican, or other), smoking status (current, former, or never), alcohol consumption (current, former, or never), physical activity level (sedentary, moderate, or vigorous), presence of hypertension (yes or no), diabetes mellitus (yes or no), CVD history (yes or no), and cancer history (yes or no). Our findings suggest that age and drinking status significantly influence the association between WWI and OAB risk (*P* for interaction <.05). Notably, the risk of OAB increased more significantly with higher WWI values in individuals under 50 years of age and in those who consumed alcohol. In the other subgroups, the relationship between WWI and OAB risk remained consistent.

### 3.3. Discrimination ability of different anthropometric measures

**Figure 3.**
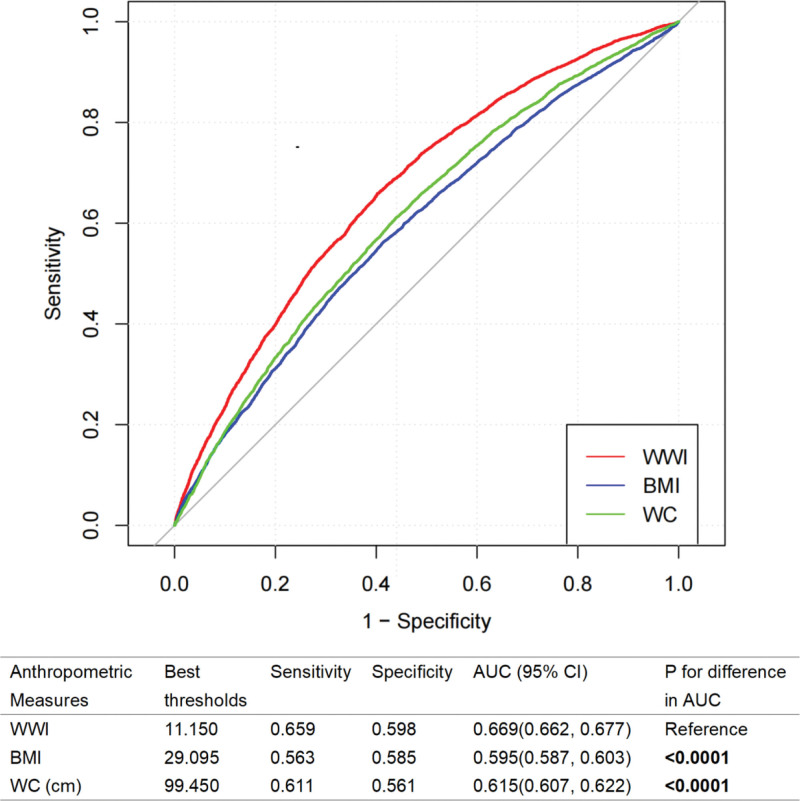
ROC curves of anthropometric indices for discriminating OAB. AUC = area under the curve, BMI = body mass index, CI = confidence interval, OAB = overactive bladder, ROC = receiver operating characteristic, WC = waist circumference, WWI = weight-adjusted-waist index.

As illustrated in Figure 3, ROC curves were generated to assess the discriminatory capacities of 3 anthropometric indices for identifying individuals with OAB, and the AUC was subsequently computed. The findings revealed that among the 3 anthropometric parameters, WWI exhibited superior diagnostic ability (*P* < .0001), with an AUC of 0.669 (95% CI = 0.662–0.677). The optimal cutoff value was determined to be 11.150, yielding a sensitivity of 0.659 and a specificity of 0.598. Furthermore, BMI and WC demonstrated AUCs of 0.595 (95% CI = 0.587–0.603) and 0.615 (95% CI = 0.607–0.622), respectively. In subgroup analyses stratified by sex, WWI consistently outperformed the other indices in both male and female groups in terms of diagnostic accuracy (Table S4, Supplemental Digital Content).

## 4. Discussion

In this study, we identified a robust association between WWI and the occurrence of OAB. Our findings from both univariate and multivariate logistic regression analyses indicate a significant correlation between higher levels of WWI exposure and an increased risk of developing OAB, even after stratifying by gender or delivery status. Furthermore, our dose–response analysis reveals a linear positive relationship between the intensity of WWI exposure and the likelihood of OAB. Subgroup analyses further showed that individuals under 50 years of age and alcohol drinkers with higher WWI exposure were more likely to develop OAB. Importantly, when employing ROC curve analysis, we observed that WWI demonstrates superior accuracy in predicting the probability of OAB compared to commonly utilized measurements such as BMI and WC.

OAB is a subset of LUTS associated with the storage phase, characterized by the presence of urinary urgency and often accompanied by other storage symptoms such as increased frequency, nocturia, and UUI. Findings from the Epidemiology of Prolific Incontinence and Continence study reveal that individuals with OAB report significantly impaired work productivity and sexual satisfaction, heightened rates of depressive symptoms and erectile dysfunction, as well as slightly diminished overall health.^[19]^

Obesity is widely acknowledged as a prominent risk factor for numerous chronic ailments, influencing the onset and progression of symptoms associated with OAB. Several studies have proposed that obesity impacts OAB through various mechanisms. One such mechanism involves the increased intra-abdominal pressure, primarily attributed to excessive central adiposity, which can exert significant pressure on the urinary bladder, leading to increased voiding frequency and urgency. A key factor in this dynamic is nerve growth factor (NGF), a pivotal protein involved in the modulation of neuronal differentiation and maintenance. Interestingly, NGF is synthesized within bladder cells. Some studies suggest that elevations in intra-abdominal pressure induce mechanical distention of the bladder, which, in turn, stimulates the secretion of NGF.^[20,21]^ A noteworthy observation from clinical studies is the increased urinary NGF expression in patients with OAB and detrusor overactivity, with a discernible correlation between NGF expression and the severity of symptoms. In a therapeutic context, symptom improvement following interventions has been accompanied by a reduction in urinary NGF levels.^[22]^

Obesity is now recognized as being closely associated with MetS and chronic low-grade inflammation.^[23]^ Insulin resistance, a significant component of MetS caused by obesity, is considered a pro-inflammatory state. Tissue inflammation leads to tissue fibrosis, which is believed to represent an inflammation-initiated, aberrant wound-healing process characterized by myofibroblast accumulation, collagen deposition, extracellular matrix remodeling, and increased tissue stiffness. These pathological conditions can affect the function of the bladder and its supporting structures, potentially leading to OAB. Multiple studies have consistently demonstrated that elevated urinary levels of inflammatory biomarkers associated with inflammation and tissue repair indicate the involvement of inflammation in OAB.^[24,25]^ For instance, patients diagnosed with OAB exhibited increased levels of inflammation-related factors and cells, including monocyte chemoattractant protein-1, CD40 ligand, macrophage inflammatory protein-1β, interleukin-12p70/p40, interleukin-5, epidermal growth factor, and growth-related oncogene-α when compared to individuals without OAB. In addition, several studies have highlighted that BMI is a significant independent factor in the detection of C-reactive protein. The AUC in predicting OAB patients was 0.55, aligning with the findings of this study.^[26]^ Furthermore, it has been observed that OAB patients with UUI exhibit higher serum CRP levels compared to those without UUI.^[27]^ Chronic inflammation is closely linked to OAB, as it leads to permanent changes and sensitization in nerve stimulation. Inflammatory factors can damage smooth muscle cells, resulting in impaired bladder function and excessive detrusor activity in obese rat models.^[28–30]^

From an alternative perspective, researchers have uncovered a significant correlation between visceral obesity (measured by WC, hip circumference, waist-hip ratio, and BMI) and the occurrence of OAB, characterized by increased urination frequency and urgency.^[31]^ Moreover, distinct gender-specific patterns in the association between various measures of obesity and OAB have been observed. In contrast to previous studies, our investigation reveals a direct link between the prevalence of OAB in women and their waist or hip circumference or BMI; however, among men, there is a decrease in the prevalence of OAB with increasing levels of obesity. Further exploration has demonstrated a positive relationship between BMI and OAB in men, up to a certain threshold.^[32]^

It is noteworthy that weight loss has demonstrated efficacy in alleviating symptoms of OAB in specific individuals, suggesting a potential modifiable risk factor for OAB.^[33,34]^ This indicates that interventions targeting weight reduction may be beneficial for individuals experiencing OAB symptoms. A randomized controlled study showcased sustained improvements in both incontinence symptoms and quality of life among women who participated in a comprehensive weight-loss program, with consistent maintenance of reduced bladder pressure due to weight loss observed for up to 6 months.^[35]^ In addition, obesity, to a certain extent, leads to limited human mobility, which may hinder an individual’s ability to reach the toilet in time, thereby causing recurrent episodes of UUI.

In clinical practice, WWI could be implemented as a screening tool to identify patients at higher risk of OAB due to central obesity. By calculating WWI, clinicians can assess abdominal fat distribution more effectively than traditional BMI or WC measurements. This could be used as part of a patient’s comprehensive assessment to help identify those who might benefit most from weight-loss interventions, such as dietary changes, physical activity, or bariatric surgery. Furthermore, WWI could be integrated into ongoing monitoring to track changes in abdominal fat and bladder function over time, helping clinicians tailor interventions for optimal outcomes. The application of WWI in this context provides an opportunity for early intervention and personalized treatment for patients struggling with OAB and obesity.

This study has both pros and cons: on the one hand, our study utilized a large and representative sample of participants from the United States, ensuring its generalizability. We comprehensively incorporated all participants and measurements spanning 13 years, enhancing the robustness of our findings. On the other hand, we employed WWI as an indicator to elucidate the impact of visceral adiposity on OAB in comparison to BMI and WC, providing valuable insights into this relationship. However, this study has several limitations that must be acknowledged. First, the diagnosis of OAB in the NHANES dataset relies solely on self-reported symptom questionnaires, which may introduce misclassification bias due to subjective interpretation and recall inaccuracy. Second, the cross-sectional design of this study limits our ability to infer any causal relationship between WWI and OAB; we can only identify associations rather than determine directionality or underlying mechanisms. Third, although ROC curve analysis demonstrates that WWI outperforms traditional anthropometric measures, its absolute discriminative ability remains modest (AUC = 0.669). This suggests that while WWI is a promising marker of central obesity, its utility as a standalone screening tool for OAB is limited. Therefore, these findings should be interpreted with caution, and future prospective cohort studies or interventional trials are necessary to validate our results, improve WWI’s predictive accuracy, and establish temporal or causal links.

## 5. Conclusion

In summary, there exists a complex interplay between obesity and OAB. A higher WWI is associated with an increased prevalence of OAB. Moreover, WWI demonstrates superior predictive potential for OAB compared to BMI and WC. Therefore, WWI may serve as a valuable clinical indicator for assessing the risk of OAB. However, further high-quality prospective studies are necessary to elucidate the underlying mechanism linking WWI and OAB.

## Author contributions

**Supervision:** Xiaoyan Hu, Tianxiao Fu.

**Writing – original draft:** Xiaoyan Hu, Weixing Jing, Yiqi Huang, Weicheng Xiao.

**Writing – review & editing:** Xiaoyan Hu, Weixing Jing, Yiqi Huang, Weicheng Xiao.

**Conceptualization:** Weixing Jing, Yiqi Huang, Tianxiao Fu, Weigang Shen.

**Data curation:** Weixing Jing, Yiqi Huang, Tianxiao Fu, Weigang Shen, Weicheng Xiao.

**Formal analysis:** Weixing Jing, Yiqi Huang, Tianxiao Fu, Weigang Shen, Weicheng Xiao.

**Software:** Tianxiao Fu, Weigang Shen.

**Funding acquisition:** Weicheng Xiao.

**Methodology:** Weicheng Xiao.

**Project administration:** Weicheng Xiao.








