# Supplementary figures and images for: Exploring the association between weight-adjusted-waist index and overactive bladder: A population-based study
Source: Medicine (Baltimore). 2026 May 8;105(19):e48763. doi: 10.1097/MD.0000000000048763 (PMC13166732; doi:10.1097/MD.0000000000048763)

**Supplementary Figure 1 The selection process of NHANES 2007 - 2020**


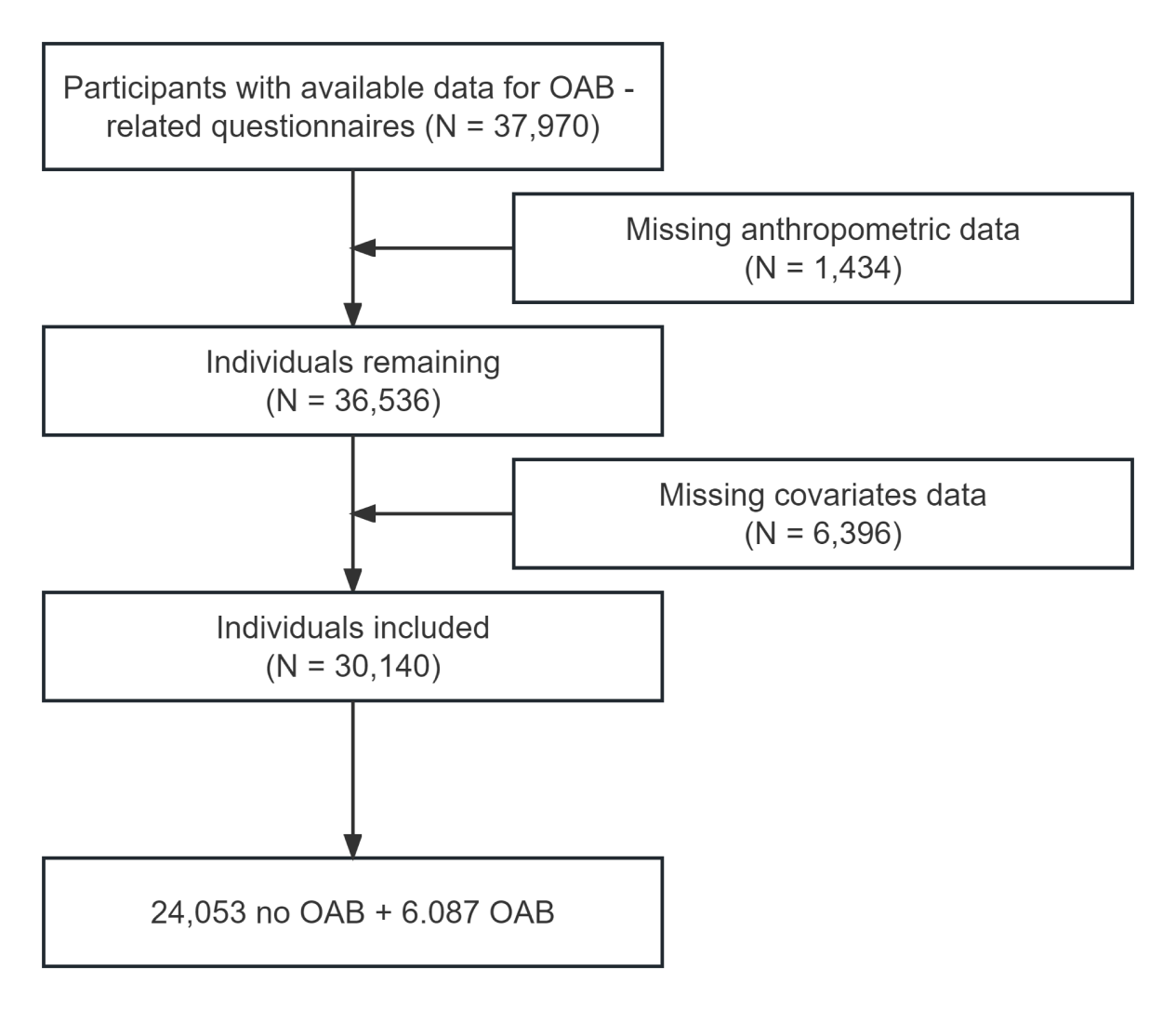

Supplement: Supplementary file 1 [file medi-105-e48763-s001.docx]
