# Supplementary material for: Exploring the association between weight-adjusted-waist index and overactive bladder: A population-based study
Source: Medicine (Baltimore). 2026 May 8;105(19):e48763. doi: 10.1097/MD.0000000000048763 (PMC13166732; doi:10.1097/MD.0000000000048763)
Supplement: Supplementary file 2 [file medi-105-e48763-s002.docx]

**Supplementary Table 1** **Criteria for Conversion of Symptom Frequencies recorded in NHANES and OABSS Scores**

| **According to NHANES Score** | **According to OABSS Score** |
| --- | --- |
| Urge urinary incontinence frequency | Urge urinary incontinence score |
| Never | 0 |
| Less than once a month | 1 |
| A few times a month | 1 |
| A few times a week | 2 |
| Every day or night | 3 |
| Nocturia frequency Nocturia score | Nocturia frequency Nocturia score |
| 0 | 0 |
| 1 | 1 |
| 2 | 2 |
| 3 | 3 |
| 4 | 3 |
| 5 or more | 3 |
| OAB was diagnosed when the total score was ≥ 3 | |

NHANES = National Health and Nutrition Examination Survey; OABSS = Overactive Bladder Symptom Score
