# Supplementary material for: Exploring the association between weight-adjusted-waist index and overactive bladder: A population-based study
Source: Medicine (Baltimore). 2026 May 8;105(19):e48763. doi: 10.1097/MD.0000000000048763 (PMC13166732; doi:10.1097/MD.0000000000048763)
Supplement: Supplementary file 3 [file medi-105-e48763-s003.docx]

**Supplementry Table 2 Logistic regression analysis of WWI and Overactive bladder by gender**

|  | Model 1 | P-value | Model 2 | P-value | Model 3 | P-value | Model 4 | P-value |
| --- | --- | --- | --- | --- | --- | --- | --- | --- |
|  | OR (95%CI) | | OR (95%CI) | | OR (95%CI) | | OR (95%CI) | |
| Male | | | | | | | | |
| WWI | 2.25(2.07,2.46) | <0.0001 | 1.72(1.54,1.92) | <0.0001 | 1.53(1.37,1.71) | <0.0001 | 1.36(1.21,1.51) | <0.0001 |
| Stratified by WWI quartiles | | | | | | | | |
| Qurrtile1 | 1 |  | 1 |  | 1 |  | 1 |  |
| Quartile2 | 1.81(1.46,2.23) | <0.0001 | 1.38(1.12,1.70) | 0.003 | 1.33(1.07,1.65) | 0.01 | 1.27(1.02,1.58) | 0.03 |
| Quartile3 | 2.81(2.25,3.51) | <0.0001 | 1.73(1.38,2.17) | <0.0001 | 1.52(1.22,1.89) | <0.001 | 1.34(1.07,1.67) | 0.01 |
| Quartile4 | 5.17(4.30,6.21) | <0.0001 | 2.81(2.24,3.52) | <0.0001 | 2.25(1.78,2.83) | <0.0001 | 1.77(1.40,2.24) | <0.0001 |
| P for trend | | <0.0001 |  | <0.0001 |  | <0.0001 |  | <0.0001 |
| Female | | | | | | | | |
| WWI | 1.98(1.84,2.12) | <0.0001 | 1.56(1.43,1.69) | <0.0001 | 1.37(1.26,1.49) | <0.0001 | 1.27(1.17,1.38) | <0.0001 |
| Stratified by WWI quartiles | | | | | | | | |
| Qurrtile1 | 1 |  | 1 |  | 1 |  | 1 |  |
| Quartile2 | 1.88(1.50,2.35) | <0.0001 | 1.47(1.16,1.85) | 0.001 | 1.34(1.07,1.69) | 0.01 | 1.29(1.03,1.61) | 0.03 |
| Quartile3 | 2.76(2.23,3.42) | <0.0001 | 1.75(1.37,2.22) | <0.0001 | 1.50(1.18,1.90) | 0.001 | 1.39(1.10,1.75) | 0.01 |
| Quartile4 | 4.73(3.89,5.74) | <0.0001 | 2.62(2.09,3.28) | <0.0001 | 1.98(1.59,2.48) | <0.0001 | 1.70(1.36,2.12) | <0.0001 |
| P for trend | | <0.0001 |  | <0.0001 |  | <0.0001 |  | <0.0001 |

Model1: unadjusted.

Model2: adjusted for age and race

Model3: adjusted for age, race, marital status, education level, PIR, recreational activity, smoking status, and drinking status.

Model4: further adjusted for creatinine urine, hypertension, diabete, CVD as well as cancer.
