# Supplementary material for: Exploring the association between weight-adjusted-waist index and overactive bladder: A population-based study
Source: Medicine (Baltimore). 2026 May 8;105(19):e48763. doi: 10.1097/MD.0000000000048763 (PMC13166732; doi:10.1097/MD.0000000000048763)
Supplement: Supplementary file 4 [file medi-105-e48763-s004.docx]

**Supplementry Table 3 Sensitivity analysis of odds of OAB after separately adjusting for delivery**

|  | OR (95CI%) | P-value |
| --- | --- | --- |
| WWI | 1.31(1.20,1.43) | <0.0001 |
| Stratified by WWI quartiles | | |
| Quartile 1 | 1 |  |
| Quartile 2 | 1.34(1.07,1.68) | 0.01 |
| Quartile 3 | 1.45(1.16,1.81) | 0.002 |
| Quartile 4 | 1.78(1.42,2.24) | <0.0001 |
| P for trend |  | <0.0001 |

Analyses were adjusted for age, race, marital status, education level, PIR, recreational activity, smoking status, and drinking status, creatinine urine, hypertension, diabete, CVD, cancer, and number of vaginal deliveries.
